# Delayed Anaphylactic Reaction to Midazolam in the Absence of Immediate Respiratory or Skin Manifestations

**DOI:** 10.1155/2023/3873076

**Published:** 2023-09-19

**Authors:** Andrew Winegarner, Mark C. Kendall, Mekhala Stephen, Afreen Siddiqui

**Affiliations:** ^1^Department of Anesthesiology, Rhode Island Hospital, Warren Alpert School of Medicine at Brown University, Providence 02903, RI, USA; ^2^Department of Interventional Pain Management and Anesthesia, Providence Veterans Affairs Medical Center, Providence 02903, RI, USA

## Abstract

Anaphylaxis, a type 1 hypersensitivity reaction, is a feared but uncommon complication of medications administered in the perioperative period. The incidence of perioperative hypersensitivity reactions has been reported to range from 1 in 20,000 to 1 in 1,361. Anesthesiologists are well aware of common causes of hypersensitivity such as paralytics and antibiotics; however, less common triggers of anaphylaxis need to be considered as well. Midazolam, a short acting benzodiazepine metabolized by cytochrome P450 enzymes, is considered very safe with a minimal risk profile. Previous reports have described adverse reactions to occur within seconds to minutes following the administration of midazolam. We describe a patient with no known history of asthma or allergies who underwent elective hydrocelectomy with spinal analgesia without incident until 42 minutes later at the conclusion of the procedure, when they experienced circulatory collapse necessitating immediate emergency treatment. This case emphasizes the necessity to improve knowledge and awareness of delayed hypersensitivity reactions following the administration of perioperative medications such as midazolam.

## 1. Introduction

Perioperative anaphylaxis is a rare life-threatening complication with the most common or causes being paralytics and antibiotics [1]. Midazolam is one of the most commonly administered benzodiazepines by anesthesiologists, primarily for anxiolysis in the preoperative period, yet the occurrence of midazolam-induced systemic hypersensitivity reported in the literature is limited [2]. Hypotension, bradycardia, a sudden drop in end-tidal carbon dioxide, a decrease in SpO_2_, and erythema have been described to occur within minutes following the administration of midazolam [3]. We present a patient with no known history of atopy who underwent elective hydrocelectomy with spinal analgesia without incident until 42 minutes into the procedure when the patient experienced circulatory collapse that required epinephrine, an emergency intubation, and a transfer to the intensive care unit (ICU). In the absence of immediate respiratory or skin manifestations, the diagnosis of anaphylaxis was not initially suspected. Increased serum tryptase levels obtained in the ICU and subsequent allergy testing confirmed midazolam-induced anaphylaxis. Our report demonstrates that anaphylactic reaction may occur anytime without the preceding classical warning signs and should not be overlooked when formulating a differential diagnosis intraoperatively. In addition, we reviewed previously published cases describing the clinical presentation of anaphylactic reactions to midazolam [4–16]. Written consent was obtained from the patient. We followed the CARE guidelines for case reports.

## 2. Case Presentation

**Figure 1:**
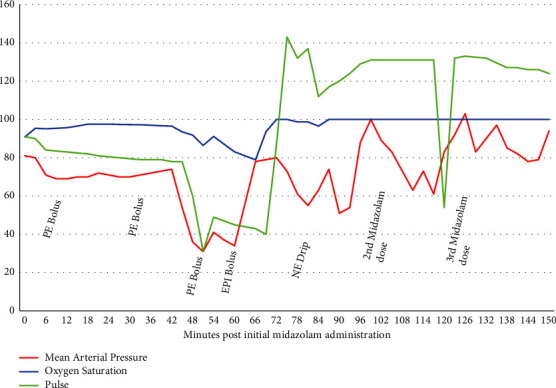
A timeline demonstrating the heart rate, mean arterial pressure, and oxygen saturation with medication administration. The first dose of midazolam was given prior to spinal analgesia in the preoperative holding area. PE = phenylephrine; EPI = epinephrine; NE = norepinephrine.

A 73-year-old, 80 kg male patient, with a past medical history of hypertension, malignant melanoma, hypothyroidism, and diabetes mellitus type 2 and a distant history of pancreatic cancer status post-Whipple procedure, was scheduled to undergo a right-sided hydrocelectomy. The patient has no personal or family history of problems with anesthesia and had received midazolam previously for his Whipple procedure. Preoperative lab and physical examination results were within normal limits with no noted allergies to any medication. After discussing the anesthetic management plan with the patient, ASA standard monitors were applied. The patient was given 2 mg of midazolam, followed by a spinal anesthetic with 3 ml of 2% mepivacaine performed at the level of the fourth and fifth lumbar vertebrae without complications. No hemodynamic changes were noted during or after the spinal anesthesia procedure. The patient did not exhibit or report any subjective changes (respiratory, cardiovascular, and skin manifestations) at that time. The patient was brought to the operating room and positioned for monitored anesthesia care with an intravenous anesthetic. Seven minutes after the placement of the initial spinal anesthetic, the patient was given 50 mcg of fentanyl and started on a propofol infusion at 60 mcg/kg/minute. 2 g of cefazolin was given shortly afterwards for antibiotic prophylaxis. The patient was kept spontaneously breathing with no airway device. Fifteen minutes into the procedure, the patient's blood pressure dropped from 108/67 to 85/61, at which point the propofol infusion rate was decreased to 40 mcg/kg/min. His blood pressures remained consistently low for the remainder of the case with systolic pressures in the 80–90 s diastolic pressures in the low 60 s, while the heart rate was stable in the low 80 s. A total of 160 mcg of phenylephrine and 10 mg of ephedrine were administered during the intraoperative period. As the case was nearing completion, 35 minutes in, the blood pressure began to decrease further which prompted another dose of 100 mcg of phenylephrine. The blood pressure continued to drop to a low of 50/29. A supraglottic airway device was placed securely with flows at 100% FiO_2_; his EtCO_2_ was 14 mmHg, his SpO_2_ at 91%, and the heart rate was 104 bpm. Two minutes later, the blood pressure dropped to 38/28 with a heart rate of 31 bpm, EtCO_2_ of 7 mmHg, and SpO_2_ of 86%. At this point, the airway was secured by placing an endotracheal tube, a second IV line was established, and 0.5 mg of epinephrine was given followed by the initiation of a phenylephrine drip at 100 mcg/min. Vitals during this ten-minute period consisted of blood pressures ranging from systolic pressures of 40–50 s, diastolic pressures of 20–30 s, a heart rate in the mid-40 s, sinus rhythm, EtCO_2_ in the 20 s, and SpO_2_ within 80–90%. Following the administration of epinephrine, the blood pressures began to increase and the phenylephrine drip was replaced with a norepinephrine drip running at 5–20 mcg/min. A timeline of the sequence of events after administration of midazolam is shown in Figure 1. The patient had received a total of 1,500 ml of lactated ringers throughout the case.

As the emergency rescue interventions were concluding, the patient was beginning to awaken while intubated, so he was given additional sedatives including a second and third dose of 2 mg of midazolam, and a dexmedetomidine infusion (0.7 mcg/kg/hr) was initiated. A triple lumen internal jugular vein catheter was placed successfully, and the patient stabilized on the norepinephrine drip. The intraoperative transthoracic echocardiogram revealed a hyperdynamic left ventricle and no regional wall abnormalities. Throughout the perioperative period, no urticaria, hives, rashes, or swelling were noted. No latex products were used during the case. Besides phenylephrine, there were no medications given immediately prior to the onset of circulatory collapse. There were bilateral yet diminished breath sounds, and a tachycardic heart rate and normal pulses were noted at the time of leaving the operating room.

The patient was brought to the ICU sedated and intubated where a more extensive workup was conducted. The ECG demonstrated a normal sinus rhythm, and no acute changes could be discerned on chest X-ray or point of care ultrasound examination, lowering the suspicion of a myocardial infarction or pneumothorax when contextualized with the previously mentioned physical exam findings. His electrolytes were all normal. Despite the lack of skin manifestations, or an obvious time relation to any drugs, anaphylaxis began to be suspected, so tryptase was ordered shortly after arriving to the ICU, which came back elevated (56.5 mcg/L, normal range being 0–11 mcg/L). The patient was extubated the following day and remained in the hospital for eight days until discharge. On postoperative day 7, a repeat tryptase level of 5.7 mcg/L further emphasized the acute nature of the initial reading further pointing towards anaphylaxis. On postoperative days four through seven, the patient underwent skin allergy testing for every drug administered in the preoperative and intraoperative period, and it was determined the patient had an allergic reaction to midazolam.

## 3. Discussion

Anaphylaxis is the most severe type of allergic reaction and requires emergent treatment. Anaphylactic symptoms usually present within minutes of exposure to an allergen. However, symptoms of anaphylaxis may occur half hour or longer after allergen exposure [17]. Our case is the first to report of anaphylactic reaction developing 42 minutes after allergen exposure without any preceding signs of allergic reaction.

Initially, the etiology of the patient's rapid decompensation was unclear. Differential at the time included neuraxial complication (a high spinal), respiratory impairment (pneumothorax), or cardiovascular injury (myocardial infarction). Initial lack of conventional allergic symptoms made the diagnosis of anaphylaxis less likely but was not entirely excluded. A timeline demonstrating fluctuations in the heart rate, mean arterial pressure, and oxygen saturation with medication administrations is shown in Figure 1. A drop in his blood pressures can be seen following each subsequent dosing of midazolam, albeit much less pronounced because the patient had received IV bolus of 0.5 mg epinephrine and norepinephrine infusions before the subsequent midazolam doses.

There have been 13 previous publications describing anaphylaxis to the perioperative use of midazolam over the past 30 years [4–16]. An overview of this case together with earlier published reports of perioperative anaphylactic reactions regarding midazolam is presented in Table 1. The median time to the onset of symptoms of anaphylaxis was 2 minutes (1–7.5). Approximately, 85% of the reported cases presented with generalized skin symptoms. Kim et al. described a case in which a 59-year-old male scheduled to undergo an orthopedic procedure presented with urticarial skin rashes of the upper and lower extremities 30 minutes following the administration of midazolam [9]. The serum tryptase level was measured confirming anaphylaxis. Our case was different in that the patient did not immediately present with any skin manifestations, swelling, or apparent respiratory problems, and circulatory collapse did not occur until 42 minutes after the administration of midazolam.

When elevated tryptase levels confirmed anaphylaxis to be the reason of the patient's decompensation, perioperative administration of cefazolin or mepivacaine was presumed to be the cause. Midazolam was not even considered until the skin allergy testing results came back. Even though elevated levels of tryptase are associated with the diagnosis of anaphylaxis, only 38% of previously reported cases obtained serum tryptase levels. Our patient fully recovered without any other adverse events. The patient was informed to strictly avoid the use of midazolam in the future.

Our case demonstrates variability in anaphylaxis presenting symptoms and duration of time from administration of the allergen to full anaphylactic reaction. It emphasizes the importance to evaluate all perioperatively administered medications and not just the common offending agents.

## Figures and Tables

**Table 1:** The current together with previously reported anaphylactic perioperative reactions involving midazolam.

Author	Age	Sex	Wt (kg)	Surgery	Midazolam mg	Prior exposure	Onset of symptoms (min)	Epinephrine (mg)	H2 blockers	Steroids	Flumazenil (mg)	Erythema	Tryptase (*μ*g/L)	Skin testing
Winegarner et al.	73	M	80	Hydrocelectomy	2	Yes	42	0.5	No	No	No	No	56.5	Yes

Nucera et al. [4]	54	M	NA	Cholecystectomy	2	NA	“Few minutes”	2	No	No	No	No	NA	Yes

Jeon et al. [5]	62	F	46	Discectomy	1	Yes	1	0.1	Ranitidine 100 mg	Dexamethasone 5 mg	0.25	Yes	NA	Yes
										Methylprednisolone 125 mg				

Çakmakcı et al. [6]	17	M	73	Bone marrow aspiration	5	Yes	5	0.5	No	Methylprednisolone 40 mg	No	Yes	NA	NA

Landsem et al. [7]	17	M	87	EP ablation	2	No	10	“Yes”	No	NA	No	Yes	31.7	Yes

Bernardini et al. [8]	7	M	NA	Adenoidectomy	0.5 (PO)	No	Immediate	No	No	Betamethasone 4 mg	No	No	NA	Yes

Kim et al. [9]	59	M	68	Ankle ORIF	3 (IM)	Yes	30	0.5	Pheniramine 40 mg	Methylprednisolone 250 mg	No	Yes	29.9	Yes
										Hydrocortisone 100 mg				

Shin et al. [10]	53	F	60	EGD	5	NA	5	1	NA	Dexamethasone 5 mg	0.5	Yes	14.8	No

Ayuse et al. [11]	59	F	56	Mandible tumor resection	5	NA	Immediate	0.05	No	Methylprednisolone 1000 mg	No	Yes	NA	NA

Shrivastava [12]	37	M	68	Elbow surgery	2	No	2	0.1	Ranitidine 150 mg	Hydrocortisone 200 mg	No	Yes	3	Yes

George and Williams [13]	23	M	53	Lymph node biopsy	1	NA	2	0.05	Ranitidine 150 mg	Hydrocortisone 100 mg	No	Yes	2	Yes

Hwang et al. [14]	39	M	65	Endoscopic pansinus	2	NA	Immediate	No	NA	NA	No	No	NA	Yes

Uchimura et al. [15]	26	F	NA	NA	2	NA	15	Yes	NA	NA	No	Yes	NA	NA

Fujita et al. [16]	38	M	NA	Cervical fusion	10	NA	Immediate	0.05	NA	Methylprednisolone 1000 mg	No	Yes	NA	No

EP, electrophysiology; EGD, esophagogastroduodenoscopy; ORIF, open reduction and internal fixation; PO, per orally; IM, intermuscular; NA, not specified in the article; doses are specified in the table if they were provided in their respective reports. Allergy testing performed weeks after discharge. Normal range of tryptase is 0-11 *μ*g/L.

## Data Availability

The data used to support the findings of this case report are included within the article.
